# Supplementary material for: Are carbon emissions trading and green financial instruments synergistic? -Comprehensive quantitative research based on content analysis
Source: PLoS One. 2024 Mar 7;19(3):e0298601. doi: 10.1371/journal.pone.0298601 (PMC10919601; doi:10.1371/journal.pone.0298601)
Supplement: S1 Data — (ZIP) [file pone.0298601.s001.zip › Data/地级市Z_F工作报告.zip!/┴╔─■/╙¬┐┌╩╨/└┤╫╘╣1⁄2╓┌║┼í╛┬φ┐╦╩2╛▌═°í┐_2013.docx]

各位代表：

　　现在，我代表市政府向大会报告工作，请予审议，并请市政协委员和列席会议的同志提出意见和建议。

　　一、2012年和过去五年工作的回顾

　　过去的一年，在省委、省政府和市委的正确领导下，在市人大、市政协的监督支持下，市政府组织带领全市人民，积极应对严峻复杂的经济形势，奋力拼搏，开拓进取，全市经济社会保持平稳较快发展。

　　预计全年地区生产总值完成1390亿元，增长11%；固定资产投资1100亿元，增长26%；社会消费品零售总额340亿元，增长16%；公共财政预算收入170亿元，增长21.3%；城镇居民人均可支配收入24028元，增长15%；农民人均纯收入12475元，增长17%。

　　（一）沿海经济带开发建设快速推进

　　招商引资和项目建设实现新突破。扎实开展“重大项目推进年”活动，招商引资成果显著。实际利用外商直接投资12亿美元，增长10%；内联引资468亿元，增长30%。开工亿元以上项目750个。投资215亿元的天盛重工一期、投资100亿元的恒力聚酯一期等一批项目竣工投产；一期投资151亿元的正威国际聚酰亚胺、投资50亿元的北方重工装备制造、投资26亿元的沈鼓大型装备等一批项目开工建设；投资70亿元的修正药业大型物流基地、投资30亿元的再生资源废金属加工和物流基地等一批项目正式签约。

　　港口发展再上新台阶。规划建设了8.25平方公里的营口港物流园区，现代物流服务中心主体完工。港口金融创新试验区经省政府批准设立。港口货物吞吐量完成3亿吨，增长15%；集装箱运量480万标箱，增长19%。

　　重点园区发展迈出新步伐。开发区承载能力不断提升，开工亿元以上项目147个，主要经济指标增幅均达20%以上。北海新区电机产业园创业中心、服务中心、检测中心和标准化厂房投入使用，累计开工千万元以上电机项目93个。产业基地开工千万元以上项目200个，新增投产企业60户，二期达到项目入驻条件；中小企业创业园新增投产企业51户，一期南扩工程全部完成。高新区新增高新技术企业13户，进入国家"863"计划及科技支撑计划项目6个，出库3个。仙人岛能源化工区公共财政预算收入等主要经济指标增幅均达40%以上。沿海新兴产业区新增投产企业25户。

　　外贸出口完成37.5亿美元，增长15%。新增出口企业98户，8个出口基地入围全省百强。

　　（二）工业经济保持健康发展

　　狠抓以企业为核心的经济运行。面对经济下行压力，加大企业帮扶力度，出台支持中小微企业发展的意见和配套政策，逐一解决重点企业运营、重点项目建设中的困难和问题，工业经济实现企稳回升。规模以上工业增加值实现770亿元，增长12.5%；新增规模以上工业企业113户，达到1422户。

　　产业集群发展壮大。17个工业产业集群实现销售收入2537亿元，增长22%。其中，镁产品及深加工产业集群规模以上企业达到205户，实现销售收入720亿元，增长24%；石化产业集群规模以上企业达到129户，实现销售收入400亿元，增长43%。汽保、乐器等产业集群销售收入增幅均达20%以上。

　　高新技术产业快速发展。卓异装备制造、新东北电气等一批高科技企业成长壮大，大族冠华等7户企业成功并购日本筱原株式会社等8户海内外科技型企业，实现低成本扩张。高新技术产品增加值实现288亿元，增长20%。

　　（三）农业和县域经济稳步发展

　　粮食生产获得好收成，总产量达14亿斤；农业增加值实现111.5亿元，增长5%。

　　现代农业发展水平不断提高。新建设施农业小区1.8万亩，新增“三品”认证面积10.7万亩，认证率达82%，居全省第3位。新增省级以上农产品品牌29个，达到83个，居全省第2位。4.3万亩节水滴灌工程全面实施。新建、续建北方糖业等投资10亿元以上重大农产品加工项目6个。农产品出口创汇1.35亿美元，增长31%。

　　县域经济增势强劲。县均地区生产总值、固定资产投资、公共财政预算收入分别增长11%、30%、26.4%。加大东部山区扶贫开发力度，贫困乡镇农民人均纯收入增加1080元。

　　（四）服务业和城区经济发展呈现强劲态势

　　服务业增加值实现528亿元，增长11%，占地区生产总值比重达到38%。

　　服务业集聚区建设取得新成果。新增省级现代服务业集聚区3个，总数达到6个。营口万达商业广场、大石桥兴隆大家庭摩尔世界等一批城市综合体开工建设。

　　旅游业发展实现新突破。旅游总收入185亿元，增长25.2%。忆江南温泉谷等一批重大温泉旅游项目投入运营，“中国北方最佳温泉城”影响力进一步扩大。推出营口主城区和辽河西大街旅游组团，域内完整的旅游链条正在形成。

　　房地产业健康发展。建立外来人口服务中心和购房接待展示中心，采取房交会、扩大货币化安置比例等措施，保持了房地产市场基本稳定。

　　现代服务业快速发展。成功举办中国（营口）第七届汽保博览会、中国（营口）国际乐器博览会和营口第四届农博会，参展企业和销售额均创历史最好水平。大宗商品交易市场投入运营。银行业金融机构本外币存款余额达1300亿元，贷款余额达1145亿元，分别增长17%和17.5%。

　　（五）城市建设和环境保护进一步加强

　　高水平编制城市规划。《营口市城市总体规划》、《营口市三个一体化发展规划》和《营口市主城区控制性详细规划》编制完成。

　　城市基础设施不断完善。机场项目得到国务院和中央军委批复，机场跑道基础工程基本完成，航站楼开工建设；高铁营口东站、盖州西站、鲅鱼圈站投入运行；500千伏南海输变电等电网工程竣工；市府路、得胜路等15条道路改造和镜湖公园改造等重点工程全部完工。

　　城市管理全面加强。出台了《关于加强城市管理的意见》等一系列制度措施，建立起市、区城市管理联动机制。

　　“清洁营口”13个专项整治战役成效显著，城市面貌明显改观。城市“绿叶杯”竞赛成绩居全省前列，产业基地明湖广场、白沙湾黄金海岸获省城市绿化、美化、亮化、净化范例奖。

　　生态环境明显改善。完成造林35.1万亩，新增城市绿地3600亩；“大浑太”流域污染治理13项工程在全省率先竣工运行；51个村农村环境连片整治工程全部完工；完成矿山治理2135亩，“青山工程”取得阶段性成果。

　　（六）民生和社会事业全面进步

　　民生保障进一步加强。15件重点民生工程完成年度计划。新增实名制就业7.9万人，城镇登记失业率为2.1%，比上年下降0.7个百分点。企业基本养老保险扩面新增2万人；城镇基本医疗保险扩面新增7.6万人。工伤、失业等险种的保障标准全面提高。新农保和城镇居民养老保险覆盖率达到90%，城乡低保实现应保尽保。

　　社会事业不断发展。义务教育均衡发展示范县（区）和基础教育强县（区）工程取得阶段性成果，完成大学园申办本科院校评估工作。文化惠民工程全面实施，乐器产业园成为全省首家重点文化产业基地。启动县级公立医院改革试点，13家乡镇卫生院实施了基本药物制度。稳定低生育水平，人口出生率、自然增长率分别为5.82％。和1.22％。。“十二运”场馆建设基本就绪。

　　（七）民主法制建设不断强化

　　自觉接受人大法律监督、工作监督和政协民主监督，积极听取各民主党派、工商联、无党派人士和各人民团体的建议。人大代表建议、政协委员提案全部办复，办结率分别为78%、78.2%。认真实行重大社会公共事项决策听证制度，政务公开和科学民主决策机制不断完善。

　　深入开展“六五”普法，推进城乡警务改革，强化社会治安防控体系建设，群众安全感不断提高。严格落实安全生产责任制，安全生产形势总体稳定。认真解决群众来信来访和投诉反映的热点难点问题，维护了安定和谐的社会局面。

　　双拥共建、国防教育、民兵预备役和人民防空工作不断加强。统计、新闻出版、国家安全、民族宗教、外事侨务、史志、档案、气象、地震等工作不断强化。慈善、老龄、妇女儿童、残疾人等各项事业取得新成绩。

　　面对百年一遇的洪水袭击，全市上下众志成城，全力做好抗洪救灾和灾后重建工作，受灾群众生产生活秩序恢复正常，实现了把灾害损失降到最低和大灾之年无疫情的目标。

　　各位代表：本届政府任期即将结束。过去的五年，是营口经济实力实现历史性跨越的五年，是营口城乡面貌发生显著变化的五年，是营口人民群众生活水平提高最快的五年。

　　经济实力大幅提升，奠定了率先崛起的坚实基础。地区生产总值由2007年的552亿元，增加到1390亿元，增长1.5倍；固定资产投资由418亿元，增加到1100亿元，增长1.6倍；公共财政预算收入由37.7亿元，增加到170亿元，增长3.5倍。经济总量居全省第4位，发展速度走在全省前列。

　　产业结构不断优化，促进了发展方式转变。三次产业结构由2007年的9.7∶54.5∶35.8调整到7.9∶54.1∶38。农业现代化水平快速提升，设施农业由2007年的15万亩，发展到20.4万亩；农业机械化综合水平由2007年的51%，提高到69.8%；水稻平均单产和最高亩产连续四年居全省首位，连续四年夺得省农田基本建设“大禹杯”，连续九年无重大动物疫情发生。工业经济综合实力显著增强，主营业务收入超亿元工业企业由2007年的180户，发展到550户；高新技术产业增加值比2007年增长5.2倍，占地区生产总值的比重提高10个百分点。服务业快速发展，消费结构不断升级，社会消费品零售总额比2007年增长1.4倍。物流配送、电子商务等现代服务业呈现强劲发展态势。

　　开放型经济快速发展，形成了多点支撑的发展格局。实际利用外商直接投资比2007年增长9倍，外贸进出口总额增长2.8倍，其中出口增长2.9倍。沿海经济带累计开发土地233.6平方公里，完成投资3255.6亿元，开工千万元以上项目1634个，其中竣工投产项目910个。开发区荣获“中国最具发展潜力开发区奖”，高新区晋升为国家级高新区，中小企业创业园晋升为国家中小企业公共服务示范平台。产业基地、仙人岛能源化工区等重点园区已成为全市经济的重要增长点，百公里沿海产业带初具规模。营口正在成为国内外客商竞相涌入的投资热土。

　　“一带三轴”的城市发展格局全面拉开，城乡环境明显改善。五年城市改造总投资700多亿元，比上个五年增长近1倍。北海新区、营东新城、河海新西市、站前金湾银畔等城区建设改造快速推进，产业基地一期初具形象，鲅鱼圈区城市建设令人瞩目。五年全市新增城区面积150平方公里，城镇化率由2007年的56.6%，提高到62.2%。滨海大道、辽河特大桥、奥体中心“一场三馆”、石门水库供水工程、大伙房水库输水配套工程等一大批重大基础设施建成并投入使用；熊岳河、民兴河景观改造成效显著，成为亮丽的城市风景线。“青山工程”、“蓝天工程”和“碧水工程”全面实施，造林绿化面积比2007年增长7倍；饮用水水质显著提高，域内水体质量明显改善；城区大气优良天数位居全省首位。滨海城市带、临海景观带正在形成。

　　文化强市战略全面实施，社会各项事业不断进步。五年财政对社会事业投入155.4亿元，比上个五年增长2.9倍。坚持教育优先发展，学前教育三年行动计划成效明显，“双高普九”走在全省前列，职业教育服务经济社会发展的能力显著增强。成功创建营口大学园，结束了营口没有本科高校的历史。文化体制改革工作全国领先，公共文化服务体系不断完善，群众文化活力显著提升，广播电视人口综合覆盖率达到99%以上。医疗卫生体系建设全面加强，城乡医疗救治水平明显提高。我市连续五年荣获国家全民健身活动优秀组织奖。不断加强和创新社会管理，城乡文明程度和市民文明素质不断提高，我市进入创建全国文明城市工作先进市行列。

　　倾力实施民生工程，人民生活明显改善。五年新增就业42.5万人次，养老、失业保险金发放标准分别比2007年提高90%和275%，城镇职工基本医疗保险参保人员增加17.9万人，启动实施了城镇居民基本医疗保险，参保覆盖面达到95%。城市人均居住面积由2007年的24.2平方米，提高到31.8平方米。主城区集中供热面积达到2260万平方米，比2007年增长38%。主城区实现24小时供水，结束了百年来间歇性供水的历史。投资2.6亿元，解决了49.7万农村人口的饮水安全问题。五年减少贫困人口11.9万人，连续三年荣获省政府扶贫开发优胜奖。城镇居民人均可支配收入和农民人均纯收入分别比2007年增长97.9%和115.4%。

　　这些成就的取得，得益于我们牢牢抓住国家实施东北振兴、辽宁沿海经济带和沈阳经济区发展战略的重大历史机遇，把中央的战略部署变成营口振兴发展的生动实践；得益于我们认真贯彻落实省委、省政府一系列重大决策，走出一条符合营口实际的发展道路；得益于我们始终坚持发展第一要务不放松，建立健全充满活力的新体制、新机制；得益于我们把人民幸福作为工作追求，激发了全市干部群众昂扬向上的工作热情，形成了万众一心谋发展的强大合力。

　　各位代表：营口的发展进步来之不易。这是在改革开放多年来奠定的基础上取得的，是省委、省政府高度重视、亲切关怀的结果，是市委科学决策、坚强领导的结果，是市人大、市政协有效监督、大力支持的结果，是全市干部群众团结奋斗、共同努力的结果。在此，我代表市政府，向辛勤工作在各条战线上的全市广大干部群众致以崇高的敬意！向人大代表、政协委员，向各民主党派、工商联、无党派人士和人民团体，向中省直单位、驻营部队、武警官兵和公安民警，向所有关心、支持营口发展的各界人士表示衷心的感谢！

　　在看到成绩的同时，我们也清醒地认识到，经济社会发展和政府工作还存在一些问题。经济总量小、大型企业少、产业结构单一、发展方式粗放的状况还没有根本改变；财政支撑发展的能力不强，城乡之间、区域之间发展还不平衡；节能减排和环境保护的压力较大；城乡居民收入水平有待进一步提高，教育、医疗发展水平与人民群众的要求相比还有一定差距；政府自身建设还需加强，服务环境有待改善。这些问题，我们将采取有力措施，认真加以解决。

　　二、今后五年奋斗目标和2013年重点工作

　　各位代表：今后五年是实现营口在辽宁沿海经济带率先崛起的关键时期，是全面建成小康社会的决定性阶段。当前，世界经济低速徘徊，复苏缓慢乏力，国内经济有效需求不足，下行压力仍然很大，经济发展面临的困难和挑战仍很严峻。同时，也要充分看到我国仍处于大有作为的重要战略机遇期，辽宁正在走向又一个“黄金发展期”，营口正处于加快发展的高峰期。强劲的后发优势、丰厚的要素资源比较优势、良好的产业基础优势日益凸显；多点支撑的发展格局已经形成，一大批优质产能的释放效应正在显现；应对严峻复杂的经济形势，我们积累了经验，提振了信心，锻炼出一支具有凝聚力、战斗力的干部队伍。更为重要的是，我们有党中央、国务院、省委、省政府和市委的坚强领导，有全市上下迎难而上、奋发进取的发展激情，我们一定能够在全面建成小康社会的道路上铸就新的辉煌。

　　今后五年，全市经济社会发展的总体要求是：全面贯彻落实党的十八大精神，坚持以邓小平理论、“三个代表”重要思想和科学发展观为指导，按照市“十二五”规划和十一次党代会的总体部署，以科学发展为主题，以加快转变经济发展方式为主线，以提高经济增长质量和效益为中心，深化改革扩大开放，大力实施“以港兴市、产业立市、文化强市”战略，坚持在做大总量中调结构，在优化结构中扩增量，在加快发展中惠民生，着力增强综合经济实力，着力改善生态环境，着力提升人民生活质量和水平，为加速率先崛起，全面建成小康社会奠定坚实基础。

　　今后五年，全市经济社会发展的奋斗目标是：

　　主要经济指标继续保持较快增长。地区生产总值年均增长12%，比2010年翻一番；公共财政预算收入年均增长14%，总量突破300亿元；固定资产投资年均增长20%，总量力争达到3000亿元；社会消费品零售总额年均增长16%。经过五年的努力，全市经济总量和综合竞争力再上新台阶。

　　现代产业发展新体系基本建立。工业化、信息化、城镇化、农业现代化同步推进，三次产业和城乡之间、区域之间协调发展；传统产业转型升级，新兴产业快速发展。“458”工业产业集群发展水平全面提升，11个服务业集聚区基本建成。科技进步对经济增长的贡献率达到60%以上，营口步入创新驱动、内生增长的发展轨道。

　　开放型经济发展水平大幅提升。全力推进港区港市联动，加快沿海经济带开发开放步伐，构建港口与腹地紧密互动，港口、物流、产业、城市四位一体的发展格局。实际利用外商直接投资、外贸出口年均分别增长10%。港口货物吞吐量年均增长10%，达到4.5亿吨以上；集装箱运量年均增长11%，达到800万标箱以上。建立起互利共赢、多元平衡、安全高效的开放型经济体系。

　　滨海、带形、组团、生态型特大城市基本建成。深入实施“一带三轴”城市发展规划，大石桥与老边、盖州与北海、鲅鱼圈与仙人岛实现一体化发展。加快城镇化进程，县城建设、小城镇发展全面提速；城市基础功能和承载力不断提升，城市规模进一步扩大；资源节约型、环境友好型社会建设取得重大进展，生态文明建设更加完善，引导全社会更加珍爱自然，保护环境，建设山清水秀的美丽营口。

　　人民生活水平显著提高。城乡居民人均收入年均增长13%，比2010年翻一番。加快推进基本公共服务均等化，基本实现教育现代化，办好人民满意教育；人人享有基本医疗卫生服务；全面建成覆盖城乡的社会保障体系，基本建成住房保障体系，人民群众安居乐业，社会和谐稳定。

　　各位代表：五年的目标振奋人心，五年的任务艰巨而繁重。实现这样的目标，需要我们加大港区港市联动力度，推进新老城区一体化发展，加速产业和人口集聚，全面提升城市发展水平；需要我们加快创新体制机制，充分激发每一个区域、每一个经济单元的发展活力，进一步增强多点支撑的能力；需要我们以更大的魄力、更强的举措、更扎实的作风，使每一项工作、每一个项目都能够高效推进，让实实在在的发展成果惠及全市人民。

　　2013年，是贯彻落实党的十八大精神的第一年，也是新一届政府履职的开局之年。要按照省、市委十一届五次全会的总体部署，扎实做好今年的各项工作，加快推进营口在辽宁沿海经济带建设中率先崛起。

　　全市经济社会发展的主要预期目标是：地区生产总值增长11%；固定资产投资增长25%；公共财政预算收入增长13%；社会消费品零售总额增长16%；城镇居民人均可支配收入增长15%；农民人均纯收入增长17%。

　　（一）加大招商引资和项目建设力度

　　继续开展“重大项目推进年”活动。重点实施“四个百项”工程，即百项工业结构调整项目、百项服务业项目、百项基础设施项目、百项高技术项目。其中，亿元以上项目331个；竣工投产项目98个，加快建设项目97个，新开工项目131个，签约储备项目74个。总投资5256亿元，当年完成投资1143亿元。

　　集中精力抓招商。围绕主题概念，瞄准重点国家和地区，加大招商引资力度。全市实际利用外商直接投资13.2亿美元，增长10%；内联引资608亿元，增长30%。加快发展对外贸易，外贸出口完成41.3亿美元，增长10%。

　　加快重点园区发展步伐。开发区要大力发展临港产业，强力推进南部新区一、二期开发，开工亿元以上项目110个，固定资产投资增长25%以上。北海新区要加大服务业集聚区和电机产业园建设力度，开工千万元以上项目85个，固定资产投资增长30%以上。产业基地要全面提升开发建设水平，开工千万元以上项目220个，投产企业达到170户；中小企业创业园开工千万元以上项目50个，投产企业达到180户。高新区要加快渤海科技城建设，开工科技研发、新兴产业项目30个，千万元以上项目55个，主要经济指标增幅达到30%以上。仙人岛能源化工区开工百亿元以上项目2个，固定资产投资和公共财政预算收入增幅达到50%以上。沿海新兴产业区开工亿元以上项目30个，投产企业达到104户。

　　强化要素保障。坚持集约节约用地，加大闲置土地清理力度，抓好增减挂钩、填海造地，充分使用“三类”用地，保障项目用地需求。坚持多渠道融资，抓好上市融资、银行信贷融资，利用企业债券、信托、融资租赁、风险投资等多种金融工具，保证项目资金需求。

　　（二）全面提升工业经济综合实力

　　继续实施企业提升工程。全市新增规模以上工业企业100户，新增销售收入超10亿元工业企业5户，规模以上工业增加值增长14%。

　　努力促进冶金、石化、装备制造、镁质材料、纺织服装、新型建材六大支柱产业快速健康发展。加大对现有企业的扶持力度，加快老企业技术改造，引导企业开拓市场，搞好产品销售；加强企业信息化建设，促进“两化”融合，不断提高管理水平和产品档次；支持企业加大合资合作力度，实现规模快速扩张。

　　加快推进五矿技改H钢、天盛重工、北钢管业、中船重工等10个重点项目建设，拉长钢铁产业链，优化产品结构，提高钢铁及深加工产业集群发展质量。

　　加快推进东吉集团镁功能材料、新发展集团硅钢级氧化镁、博尔凯特镁合金压铸件、银河镁铝公司镁合金板等40个重点项目建设，提升镁产品及深加工产业集群发展水平。

　　加快推进正威国际聚酰亚胺一期、林氏化学增塑剂、辽宁绿地国丰C9深加工等60个重点项目建设，着力打造中国北方精细化工产业基地，实现石化产业集群化发展。

　　加快推进辽宁防爆电机、美国IES集团电机控制保全系统、辽宁华电电器公司变频调速电机等60个重点项目，电机产业园新开工项目50个，投产企业40户。

　　以拥有自主知识产权、掌握核心技术为切入点，发展优势产品，壮大优势企业，打造中国汽车保修装备产业基地和世界汽车保修装备制造中心；加快乐器产业园建设，大力发展传统优势乐器，培育壮大龙头企业，塑造乐器之都新形象。

　　加快沈鼓集团、北方重工、无限极等一批重点项目建设，进一步抓好输变电设备、汽车及零部件、铝制品及深加工、工程塑料制品、管件阀门、食品饮品等产业集群发展，促其尽快做大做强。新增销售收入超百亿元产业集群3个，钢铁及深加工和石化产业集群销售收入超500亿元。

　　认真做好43户重点用能企业节能降耗工作，淘汰铁合金、印染、水泥等行业的落后产能。万元工业增加值能耗降低率达到3%以上。

　　（三）加快发展现代农业和县域经济

　　农业增加值增长5%。县域生产总值、公共财政预算收入、固定资产投资分别增长17%、20%、27%，确保完成县域经济倍增计划。

　　积极发展现代农业。努力创建优质水稻高产示范县，争创省设施水果和蔬菜示范区。新建设施小区1万亩，新增“三品”认证面积10万亩、订单农业10万亩、水产品工厂化养殖3.4万平方米。抓好“菜篮子工程”，新增蔬菜基地1.5万亩。争创省以上农产品品牌10个。新增节水滴灌面积3.3万亩。加大农业机械化推广力度，实现水稻生产全程机械化。

　　加快农业产业化步伐。提升盖州苹果和大石桥南果梨的规模和品质，做大做强优势特色产业。津大生态肥料等6个投资10亿元以上重大农产品加工项目要全部建成投产。抓好农产品出口基地建设，打造出口农产品质量安全示范区。

　　深化农村改革。加快推进土地承包权流转，完善土地（林地）经营权流转市场建设。继续开展宅基地和林权抵押贷款、设施农业产权认证等试点工作，破解农业融资难题。

　　深入实施东部振兴战略。加大投入力度，确保东部山区农民人均纯收入增加1200元。

　　（四）进一步提高服务业和城区经济发展水平

　　服务业增加值增长11%，占地区生产总值比重达到39%。

　　加快建设中央商务区，重点推进营口万达商业广场、台湾名品城等10个项目。规划建设鲅鱼圈海滨温泉、营东新城现代物流、大石桥“龙街”等11个服务业集聚区。大力发展现代物流、电子商务、服务外包、会展等现代服务业态。加快金融业发展，引进外埠银行业金融机构2家。

　　大力发展旅游产业。加快推进万科海港城、北方亚龙湾温泉城、奕丰·泉天下等一批重大项目建设，完善鲅鱼圈石家沟画家村、双凤山旅游度假村等一批乡村旅游景点，积极发展沟域旅游，努力把白沙湾黄金海岸建设成为国家AAAA级景区；加强辽河西大街建设管理，完善配套服务，打造中国历史文化名街。旅游总收入实现226亿元，增长22%。

　　努力扩大消费需求。加强城乡流通体系建设，培育商贸流通龙头企业，巩固“万村千乡”市场建设成果，培育开发“夜经济”等消费热点。建立15家平价商店，加强价格监管和市场监测，保障市场供应，保持居民消费价格基本稳定。

　　（五）着力推进科技创新

　　高新技术产品增加值增长25%，占地区生产总值比重提高2个百分点。

　　启动创新型企业工程。组织10户有实力、有潜力的大企业，并购一批国内外科技型企业，引进先进技术，培育创新型龙头企业。培育100个产品科技含量高、发展前景好的创新型中小企业，实施100个技术创新项目和5个引进技术消化吸收再创新项目。

　　加强产学研合作。推进与省内20所大学的战略合作，支持企业参与科技研发和成果转化。实施20个重大科技创新和产品研发项目，加快大石桥国家可持续发展实验区建设。积极引进和培养科技领军人才、新兴产业紧缺人才和高技能人才。

　　加大科技创新投入力度。安排5000万元资金，支持科技创新工作。在高新区、产业基地、中小企业创业园、鹊鸣湖科技产业园建立种子基金，支持初创型企业加快技术创新。鼓励保险公司、再保险公司、担保公司等机构共同参与科技项目风险管理，为科技型中小企业提供融资担保。

　　（六）加快城镇化步伐

　　加强城乡规划、建设和管理。全面落实“三个一体化”发展规划。以打造站前金湾银畔和河海新西市为目标，加快北部、西部老城区改造步伐。创新城市管理体制机制，构建“大城管”格局。实施“智慧营口”建设，促进城市信息化管理提档升级。进一步加强市容市貌、道路交通、建筑工地管理，提高城市精细化管理水平，努力创建全国卫生城市。

　　着力推进县城建设。大石桥市要抓紧“镁都新城”和高铁站建设，加快金水湖和淤泥河等水系改造进度，高标准推进老城改造。盖州市要加快推进“盖州-北海一体化”发展，高水平推进大清河景观改造，确保兴隆集团商业综合体、闽南建材城等项目年内投入运营。

　　促进房地产业平稳健康发展。以工业地产、商业地产和旅游地产为重点，有序推进房地产开发。引进知名品牌企业，提升房地产档次和品位。加大城中村、城边村改造力度，推动城镇基础设施和公共服务向农村延伸。房地产投资和商品房销售额均增长15%以上。

　　加快推进人口集聚。进一步完善政策，加大城市推介力度，积极吸引外地人来营置业安居。继续抓好列入省规划的7个区镇和6个村的城镇化试点工作，有序推进农业人口向城镇转移，稳妥解决进城农民户籍、就业、住房、社会保障等问题，推动人口集聚规范化、常态化。

　　（七）抓好基础设施和生态环境建设

　　完善城市基础设施。加快兰旗机场建设，力争年底实现通航。加强港口集疏运能力建设，确保港口货物吞吐量达到3.3亿吨，增长10%；集装箱运量530万标箱，增长10.5%。高铁客专快速通道、营口汽车客运站改造等重点交通工程确保年内完工。完成华能仙人岛热电厂一期和城市生活垃圾处理厂及配套、东部污水处理厂中水回用工程。实施大清河、大旱河、咸水河整治和公园、广场照明改造及16条街路绿化提升工程。开工建设市图书馆、档案馆和市民活动中心。

　　加强生态城市建设。实施造林11.6万亩、义务植树350万株、森林抚育1.2万亩，争创全国森林城市。深入实施“青山工程”，对高铁、高速公路和铁路两侧山体进行全面修复。全面实施“蓝天工程”，加快城区集中供热和“一县一热源”建设；加大企业脱硫脱硝工程推进力度和“黄标车”淘汰力度，进一步提高大气质量。实施“碧水工程”，新建3座乡镇污水处理厂，完成营口西部污水处理厂提标改造。推进42个村农村环境连片整治，努力创建生态示范乡镇和示范村。万元GDP能耗下降3.77%。

　　（八）大力发展民生和社会各项事业

　　坚持倾情倾力改善民生，让人民群众的生活更加殷实、更加幸福。完成上年结转的3件重点民生工程，新启动10件重点民生工程。

　　加强就业和社会保障体系建设。全年新增实名制就业6.5万人，城镇登记失业率确保2.8%以内。保持城镇零就业家庭动态为零，确保困难家庭高校毕业生全部就业。统筹推进城乡保障体系建设。做好养老、医疗等保险扩面和基金征缴工作，提高城镇居民基本医疗保险和新农合的补助标准及城乡低保保障标准，新农合参合率稳定在96%以上。

　　大力发展社会事业。推进各级各类教育健康发展，高标准建设营口市中等职业教育园区，全力推进营口理工学院申办和建设工作。继续开展文化惠民工程，丰富群众文化生活；加快发展文化产业，打造一批具有较强影响力的文化品牌。深化医药卫生体制改革，全市乡镇卫生院和村卫生室全部实施基本药物制度；实施城乡居民公共卫生服务工程，完善基层公共医疗卫生服务体系；建立健全食品安全溯源机制，实行严格的食品药品安全监管制度。认真做好人口和计划生育工作，不断提高出生人口素质。广泛开展群众体育运动，认真做好“十二运”营口赛区赛事承办工作。

　　努力创新社会管理。强化基层社会管理服务平台建设，完善社区就业、卫生、治安等管理和服务功能，全面提高社区网格化管理水平。加强对特殊人群的教育、管理和服务，从源头减少不稳定因素。坚持以群众工作统揽信访工作，切实解决群众合理诉求，维护社会公平正义。

　　加强“平安营口”建设。深入开展“六五”普法活动，加强社会治安综合治理。深入推进城乡警务改革，进一步完善立体化社会治安防控体系，依法严厉打击违法犯罪。推进安全生产“标准化”建设，加大安全生产监管和专项检查力度，坚决遏制重特大事故发生。健全应急救援体系，提高突发事件处置和防灾减灾能力。

　　加强国防教育和民兵预备役建设，做好人民防空工作，积极开展双拥共建活动。进一步做好统计、新闻出版、国家安全、民族宗教、外事侨务、史志、档案、气象、地震等工作。积极发展慈善、老龄、妇女儿童、残疾人、未成年人等各项事业。

　　（九）加强政府自身建设

　　坚持依法行政，努力提高决策水平。自觉接受人大及其常委会的法律监督和工作监督，支持政协履行政治协商、民主监督和参政议政职能。加强行政监察、审计监督，重视群众监督、舆论监督。依法开展行政复议、行政应诉工作，保障公民与法人的合法权益。强化政务公开，坚持重大事项集体决策、专家咨询和听证制度，做到科学决策、民主决策。

　　继续解放思想，增强攻坚克难能力。面对复杂形势和艰巨任务，各级政府和全体公务员必须勇于开拓、敢于担当，以昂扬的精神状态、创新的工作举措，积极化解各种困难和风险。更加注重吸收先进地区的新理念、新做法，注重发掘人民群众在实践中创造出的新经验，认真总结，大胆实践。

　　进一步转变作风，切实加强软环境建设。认真执行中央提出的八项规定，规范政务活动，切实改进会风、文风，树立良好政风。要加大软环境的整治力度，坚决纠正庸懒散奢等不良风气，着力提高办事效率和服务水平。要大力弘扬脚踏实地、严谨求实的工作作风，对各项工作任务要一件一件抓落实，一项一项抓成效，不断提高执行力和公信力；牢固树立“过紧日子”思想，最大限度压缩一般性支出，严禁铺张浪费；要加强调查研究，密切联系群众，认真解决好人民群众关注的热点难点问题。加快转变政府职能，进一步提高政府工作水平。坚持从严治政，积极推进惩治和预防腐败“五大系统”建设，努力建设清廉政府。

　　各位代表：站在新起点、实现新发展、开创新局面，是人民寄予的历史重任，是历史赋予的庄严使命。让我们紧密团结在以习近平同志为总书记的党中央周围，在省委、省政府和市委的坚强领导下，紧紧依靠全市人民，拼搏进取，扎实工作，共同创造小康社会的美好生活！
